# Effects of requested, forced and denied shift schedule change on work ability and health of nurses in Europe -Results from the European NEXT-Study

**DOI:** 10.1186/1471-2458-13-1137

**Published:** 2013-12-05

**Authors:** Michael Galatsch, Jian Li, Hanne Derycke, Bernd Hans Müller, Hans Martin Hasselhorn

**Affiliations:** 1 Chair of Family Nursing and Community Care, Department of Nursing Science, Witten/Herdecke University, Stockumer Str. 12, D-58285 Witten, Germany; 2 Institute of Occupational and Social Medicine, Center for Health and Society, Faculty of Medicine, University of Düsseldorf, Universitätsstr. 1, D- 40225 Düsseldorf, Germany; 3 Department of Sociology, POS+, Participation, Opportunities, Structures, Ghent University, Korte Meer 3-5, Ghent B-9000 Belgium; 4 Department of Safety Engineering, Section of Occupational Safety & Ergonomics, University of Wuppertal, Gaußstr. 20, Wuppertal D-42119 Germany; 5 Federal Institute for Occupational Safety and Health (BAUA), Nöldnerstr. 40-42, D-10317 Berlin, Germany

**Keywords:** Nursing, Work schedule tolerance, General health, Work ability

## Abstract

**Background:**

Previous cross-sectional findings from the European Nurses Early Exit Study (NEXT) show that nurses who were dissatisfied with their work schedule tended to consider leaving the nursing profession. Mediating factors in this decision process may be caused by self-perceived poor work ability and/or health. The aim of this paper is to investigate changes in work ability and general health among nurses in relation to requested, forced and denied change of shift schedule.

**Methods:**

Longitudinal data from the NEXT Study was used. In total 11,102 nurses from Belgium, Germany, Finland, the Netherlands, Poland, Slovakia, France and Italy completed both the ‘basic questionnaire’ (t1) and the ’12 month follow-up questionnaire’ (t2). To examine the time-effect (repeated measures) and the group-effect of five defined groups of nurses on the Work Ability Index (WAI) and general health (SF36), an adjusted 2-way analysis of covariance (ANCOVA) was performed.

**Results:**

The nurses who wanted to, but could not change their shifts during the 12 month follow-up had the lowest initial and follow-up scores for WAI (t1: 37.6, t2: 36.6, p <0.001), lowest general health (t1: 63.9, t2: 59.2, p <0.001) and showed the highest decrease in both outcomes. Shift pattern change in line with the nurses’ wishes was associated with improved work ability and to a lesser comparatively low extent with increased decline in health scores. A forced change of shift against the nurses’ will was significantly associated with a deteriorating work ability and health.

**Conclusions:**

The findings would suggest that nurses’ desire to change their shift patterns may be an indicator for perceived low work ability and/or low health. The results also indicate that fulfilling nurses’ wishes with respect to their shift work pattern may improve their personal resources such as work ability and – to somewhat lesser extent – health. Disregarding nurses’ preferences, however, bears the risk for further resource deterioration. The findings imply that shift schedule organization may constitute a valuable preventive tool to promote nurses’ work ability and – to lesser extent – their perceived health, not least in aging nursing work forces.

## Background

The work schedule and working time are important work characteristics. Many organizations impose shift work on their employees in order to maintain productivity and permanence in an organization. The concept of shift work covers a variety of working time arrangements including working outside daytime hours (e.g. night shifts), overtime work and irregular or rotating work schedules [1]. Shift work is common in many health care organizations and nurses in particular are confronted with working in different shift systems and inflexible shift schedules which may cause unique stress and demands [2]. Earlier research has documented that shift working can be detrimental for employees’ social life leading to difficulties finding a good balance between their work and private life [3]. Moreover, a number of studies showed that an inadequate work planning and a poorly organized shift schedule may have an adverse effect on employees’ health and well-being, resulting in both less quality and quantity of sleep, poorer physical and mental health and reduced quality of performance [4]. Besides affecting employees’ self-rated health, all these adverse consequences may influence the perceptions employees have of their capabilities to cope with their perceived demands at work (i.e. work ability) [4].

But shift work also provides opportunities. The European Nurses Early Exit (NEXT) Study has shown that most nurses were satisfied with their work schedule even when working shifts. 72% of all nurses (N = 36.492, 9 countries) were satisfied with their work schedule with respect to their wellbeing and 64% with respect to their private life. Interestingly, the figures were substantially higher for nurses working “nights only” (74% and 80%, respectively; data on 6 countries, similar patterns in all countries) [5]. This may indicate that many nurses working nights only have – in the course of their professional life – chosen their preferred work pattern that best fits their individual and private needs. The findings of Oginska [6] support this: their cross sectional NEXT data analysis showed that not only the shift system itself but more importantly the discrepancy between the individual’s shift schedule preferences and his/her actual work schedule affected nurses’ intent to leave the nursing profession [6].

A desire to change shift schedule may develop due to nurses’ work/family incompatibilities but may also be due to perceived low work ability or simply because of health problems. Being denied or being allowed to change to the preferred shift schedule might thus evoke different effects on workers’ work ability and health. However, research investigating the effect of requested, forced and denied change in shift schedule on health care workers’ work ability and self-rated health is lacking.

Work ability and health are not identical concepts. This differentiation is crucial because both concepts may – when adverse – require different preventive targets and actions. Work ability refers to both individual and occupational factors that are essential to a person’s ability to cope in working life [7]. The work ability concept is based on the assumption that the ability of a worker to perform job tasks successfully depends on the equilibrium between physical and mental job demands with individual capacities, determined by health, professional knowledge and competencies, values, attitudes and motivation to work. The concept of work ability can be illustrated by the ‘house of work ability’ which consists of 4 floors where the employees’ general health constitutes the basis for work ability, the ground floor [8].

However, employees suffering from poor self-rated health do not necessarily have poor work ability [9]. In fact, Hasselhorn and colleagues [10] have found high self-reported work ability among 191 (37%) of 512 nurses with self reported very poor health. This underlines that work ability is not only a result of health and functional capacity, but may also reflect other aspects such as work demands and work organizational factors [11]. The overall question investigated in this paper is how requested, forced and denied shift schedule change affected nurses’ work ability and health.

## Methods

The European NEXT Study is a longitudinal study investigating working conditions, private life, health and future perspectives of nurses in 10 countries. Between 2002 and 2005 four self-administered questionnaires have been sent out to nursing staff (all qualification levels) in hospitals, nursing homes and home care. In total, 56,406 people from more than 600 healthcare institutions have participated. The NEXT-Study design has been described in detail in Hasselhorn et al. [5] and it was approved by the Ethical Committee of the University of Wuppertal, Germany.

This secondary data analysis was based on the longitudinal data of eight countries (Germany, Finland, France, Italy, Belgium, the Netherlands, Poland, and Slovakia). Two self-administered questionnaires with a time lag of one year were distributed among all nursing staff who remained working in the participating 552 health care institutions during the twelve months follow-up period. At baseline (autumn 2002/spring 2003; t1) 61,940 questionnaires were sent out and 34,578 were returned (overall response rate 55.8%, range from 41.3% [France] to 76.9% [Finland]). Among them, 32,008 questionnaires were with valid data for general health and work ability. Twelve months later, at follow up (t2), 55,571 questionnaires were sent and 23,523 received (response rate: 42.3%, range from 23.6% [France] to 66.3% [Finland]). An anonymous coding system was used to match the two questionnaires of each participant.

The study sample of this longitudinal study consisted of 11,102 nurses from eight countries (Belgium, n = 1,333, Germany, n = 1,696, Finland n = 2,270, France n = 859, Italy n = 1911, the Netherlands n = 1.107, Poland n = 1.469 and Slovakia n =457) who remained working in their organization during the one year follow-up and who participated in both surveys and completed both the ‘basic questionnaire’ (t1) and the ’12-months follow-up questionnaire’ (t2) (Table 1).

**Table 1:** Distribution of participants by dominant shift pattern and by country at t1 (N = 10,946, 156 missings)

**The nurses’ dominant shift pattern at t1**					
	**“Day and night shift”**	**“Shift work without night shift”**	**“Only night shift”**	**“Regular day hours”**	**N total**
Belgium	295 (22.3%)	499 (37.7%)	64 (4.8%)	467 (35.2%)	1325
Finland	1118 (51.6%)	414 (19.1%)	59 (2.7%)	574 (26.5%)	2165
France	162 (18.4%)	387 (43.9%)	138 (15.6%)	195 (22.1%)	882
Germany	763 (45.9%)	439 (26.4%)	124 (7.5%)	338 (20.3%)	1664
Italy	1010 (53.3%)	408 (21.5%)	3 (0.2%)	473 (25.0%)	1894
Netherlands	608 (54.9%)	225 (20.3%)	18 (1.6%)	249 (22.6%)	1100
Poland	999 (68.4%)	349 (23.9%)	1 (0.1%)	112 (7.7%)	1461
Slovakia	322 (70.8%)	67 (14.7%)	0 (0,0%)	66 (14.5%)	455
*Total*	*5277 (48.2%)*	*2788 (25.5%)*	*407 (3.7%)*	*2474 (22.6%)*	*10946*

### Measures

#### Shift change

Change of shifts - voluntarily or involuntarily - was measured by one question from the follow-up questionnaire (t2): “*in the last 12 months: have you changed shift pattern?*”. Shift change groups were created based on the following five answer categories: “*No, I did not ask*”, “*No, but I asked*”, “*Yes, because I asked*”, “*Yes institution imposed it*” and “*Yes, institution imposed, I accepted*”.

Consequently, the groups differentiate both work schedule change during the past 12 months as well as the nurses’ personal attitude/action in relation to the change/non-change (requested, forced and denied). The group classification does not, however, directly reflect the nurses’ general satisfaction with the work schedule nor the preferred work schedule.

#### Work ability

Work ability was assessed by the Work Ability Index (WAI). The WAI is a short questionnaire developed by K Tuomi, L Eskelinen, J Toikkanen, E Jarvinen, J Ilmarinen and M Klockars [12]. It is conceptually based on the work ability concept described above and assesses how well the workers’ resources and his/her job fit together. The highest value for the best state of work ability is 49, and the lowest work ability is 7. The WAI can be used as a screening and monitoring instrument for both, individuals and groups, within occupational health, workplace health promotion and also in scientific investigations. It has proven to be helpful in high stress level detection and prevention [13], to be a predictor for disability pension and mortality [14,15] and to be a good predictive indicator for early retirement [11]. The WAI has been translated into more than 20 languages and is highly applicable for cross-cultural comparisons [16].

#### General health

General health was measured employing a 5-item subscale which followed the suggestions of the SF36 [17]. The SF36 was constructed to survey the health status in medical outcome studies and has been designed for the use in clinical practice and research, health policy evaluations, and general population surveys. The items are to be answered on a five-point scale. For constructing the scale the original five point scale was set from 0 – 100 following the instructions of the authors [17]. One missing item per participant was tolerated for scale calculation. The Cronbach alpha coefficients of this 5-item subscale of “General health” were ranged 0.72-0.77 across the right countries in the NEXT Study.

### Statistical analyses

Descriptive analyses were used to describe the study sample at baseline. To compare the group effect of the five shift change groups on WAI and general health, an analysis of covariance (ANCOVA) was performed with adjustment for age, gender and country. The “*No, I did not ask*” group served as the reference group. This was done separately for the two outcomes WAI and general health and for t1 and t2 respectively. In addition, a general linear model (GLM) with repeated measures was used to examine the within group time effects from t1 to t2, adjusting for age, gender and country [18]. The analyses were performed by using SPSS 21.0 software.

## Results

### Study sample

The sample was composed mainly of females (84.9%). The mean age at t1 was 39.8 years (SD 9.0) with an average work experience in nursing profession of 15.3 years (SD 8.7) and an average seniority in the current institution of 8.4 (SD 7.4) years. The majority of the employees (77.3%) were registered nurses, 19.6% were assistant nurses and 3.1% had other or no nursing education. Most respondents (61.8%) worked in hospitals, 13.4% in nursing homes and 24.8% in home care services. Of all participants selected for this analysis 5,330 (47.9%) worked both day and night shifts, 2,849 (25.6%) performed shift work without nights, 412 (3.7%) worked only night shifts and a total of 2,537 (22.8%) of the nurses worked in day hours, but not shift. From all Nurses, 52.4% worked in a part time and 47,6% in a full time job.

Compared to the 20,906 nurses in the eight countries who participated at the baseline survey only (and with valid data for general health and work ability), the 11,102 nurses who participated both the baseline and follow-up surveys were significantly older (mean age: 38.6 vs. 39.3 years, p < 0.001), had a significantly lower general health (M = 65.1 vs. 63.6, p < 0.001) and lower work ability (M = 39.4 vs. 39.1, p < 0.001).

The majority of nurses in this analysis (83.0%) neither reported a change of shift nor the request of it during the past 12 months (Table 2). This group was followed by those who were forced to change shift pattern against their will (5.6%) and those who successfully had asked for a change of shift pattern (5.4%). Those who accepted a schedule change proposed by the institution were 3.6% and, finally, those who unsuccessfully had requested a change of work schedule were 2.3% of all nurses. The respective proportions varied considerably between the different countries.

**Table 2:** Participants by shift change group and country at t2 (N = 11,102)

**At t2: response to the question: “In the last 12 months: have you changed shift pattern?”**					
	**“No, I did not ask”**	**“No, but I asked”**	**“Yes, because I asked”**	**“Yes, institution imposed it”**	**“Yes, institution imposed it, I accepted”**
Belgium	1151 (86.3%)	20 (1.5%)	71 (5.3%)	60 (4.5%)	31 (2.3%)
Finland	1848 (81.4%)	54 (2.4%)	176 (7.8%)	85 (3.7%)	107 (4.7%)
France	669 (77.9%)	31 (3.6%)	73 (8.5%)	53 (6.2%)	33 (3.8%)
Germany	1391 (82.0%)	58 (3.4%)	91 (5.4%)	107 (6.3%)	49 (2.9%)
Italy	1533 (80.2%)	52 (2.7%)	74 (3.9%)	192 (10.0%)	60 (3.1%)
Netherlands	903 (81.6%)	16 (1.4%)	81 (7.3%)	37 (3.3%)	70 (6.3%)
Poland	1338 (91.1%)	22 (1.5%)	18 (1.2%)	60 (4.1%)	32 (2.1%)
Slovakia	389 (85.1%)	7 (1.5%)	12 (2.6%)	34 (7.4%)	15 (3.3%)
*Total*	*9222 (83.0%)*	*260 (2.3%)*	*596 (5.4%)*	*628 (5.6%)*	*396 (3.6%)*

### Work ability

The top lines in Table 3 show the findings for the complete sample. At t1, two groups had highly significantly (p < .001) lower work ability means than the reference group (“*No, I did not ask*”). They were the groups who – one year later – reported that they had requested a shift change (“No, but I asked” and “Yes, because I asked”). Also those whose shift change was imposed by the institution had somewhat lower WAI scores than the reference group at t1 (p < .05). At t2 the mean WAI scores of all groups of shift schedule change, apart from the group “*Yes, because I asked*”, differed significantly (p < .01 to p < .001) from the reference group “*No, I did not ask*”.

**Table 3:** Mean values for work ability and general health at baseline and one year later by shift change group, for the overall sample (top rows) and by country

			**Classification by response to the question at t2: “In the last 12 months: have you changed shift pattern?”**				
**Country**	**Outcomes**		**“No, I did not ask”**	**“No, but I asked”**	**“Yes, because I asked”**	**“Yes, institution imposed it”**	**“Yes, institution imposed it, I accepted”**
Overall		n	9222	260	596	628	396
	Work ability	t1 mean (95% CI)	39.6 (39.4 – 39.7)	37.6 (36.9 – 38.4)***	38.6 (38.1 – 39.0)***	39.1 (38.7 – 39.5)*	39.4 (38.8 – 40.0)
		t2 mean (95% CI)	39.3 (39.2 – 39.4)###	36.6 (35.8 – 37,3)*** ###	38.8 (38.3 – 39.3)	38.4 (37.9 – 38.8)** ###	40.0 (39.5 – 40.6)** #
	General health	t1 mean (95% CI)	65.3 (64.9 – 65.7)	63.9 (61.6 – 66.2)	65.2 (63.7 – 66.7)	63.8 (62.4 – 65.2)	65.6 (62.9 – 68.3)
		t2 mean (95% CI)	63.0 (62.6 – 63.4)###	59.2 (56.9 – 61.5)*** ###	64.7 (63.2 – 66.2)	60.7 (58.8 – 62.6)* ###	64.4 (62.0 – 66.8)
Belgium		n	1151	20	71	60	31
	Work ability	t1 mean (95% CI)	40.2 (39.9 – 40.5)	37.2 (34.6 – 39.7)*	38.4 (37.3 – 39.5)**	39.6 (38.4 – 40.9)	39.8 (37.5 – 42.2)
		t2 mean (95% CI)	39.9 (39.6 – 40.2)##	35.3 (33.2 – 37.3)** ###	39.2 (37.9 – 40.5)	38.6 (37.2 – 40.0)	40.1 (38.1 – 42.1)
	General health	t1 mean (95% CI)	70.2 (69.2 – 71.1)	62.3 (53.4 – 71.1)	66.5 (61.9 – 71.1)	68.9 (64.2 – 73.6)	65.2 (57.5 – 72.9)
		t2 mean (95% CI)	67.7 (66.7 – 68.6)###	55.8 (48.4 – 63.1)*	64.4 (60.0 – 68.7)	65.3 (61.2 – 69.5)	65.1 (59.6 – 70.7)
Finland		n	1848	54	176	85	107
	Work ability	t1 mean (95% CI)	39.9 (39.7 – 40.2)	38.2 (36.8 – 39.6)	39.6 (38.8 – 40.5)	38.4 (37.1 – 39.7)**	40.4 (39.5 – 41.2)
		t2 mean (95% CI)	39.8 (39.6 – 40.1)#	38.0 (36.6 – 39.4)	40.1 (39.3 – 40.8)	37.4 (35.9 – 39.0)**	40.2 (39.1 – 41.2)
	General health	t1 mean (95% CI)	66.9 (66.0 – 67.7)	64.6 (59.7 – 69.5)	69.1 (66.5 – 71.6)	64.6 (59.8 – 69.3)	66.6 (63.1 – 70.1)
		t2 mean (95% CI)	66.1 (65.3 – 66.9)#	60.7 (56.2 – 65.3)#	69.2 (66.8 – 71.6)	61.5 (56.9 – 66.2)#	65.7 (62.3 – 69.1)
France		n	669	31	73	53	33
	Work ability	t1 mean (95% CI)	38.9 (38.5 – 39.2)	38.0 (36.1 – 39.9)	38.0 (36.3 – 39.6)	39.0 (37.8 – 40.2)	37.5 (34.8 – 40.1)
		t2 mean (95% CI)	38.6 (38.1 – 39.0)	35.5 (33.3 – 37.6)** ##	37.8 (36.4 – 39.2)	37.1 (35.2 – 39.0)* ##	40.3 (38.7 – 41.8)** #
	General health	t1 mean (95% CI)	64.0 (62.5 – 65.5)	65.5 (58.6 – 72.4)	62.0 (57.1 – 66.9)	60.5 (54.2 – 66.8)	59.8 (52.0 – 67.7)
		t2 mean (95% CI)	62.2 (60.8 – 63.5)##	57.4 (50.3 – 64.6)* ##	61.7 (57.0 – 66.5)	56.4 (50.8 – 62.0)#	63.8 (57.5 – 70.1)
Germany		n	1391	58	91	107	49
	Work ability	t1 mean (95% CI)	38.1 (37.8 – 38.4)	35.5 (33.6 – 37.5)*	36.9 (35.5 – 38.3)	38.4 (37.3 – 39.4)	37.5 (35.7 – 39.4)
		t2 mean (95% CI)	37.7 (37.3 – 38.0)##	34.5 (32.6 – 36.4)*	36.6 (35.0 – 38.2)	38.1 (36.9 – 39.3)	38.6 (37.1 – 40.1)
	General health	t1 mean (95% CI)	65.1 (64.1 – 66.1)	64.2 (58.8 – 69.5)	61.3 (56.9 – 65.7)	65.4 (61.7 – 69.1)	64.9 (59.5 – 70.3)
		t2 mean (95% CI)	63.9 (62.9 – 64.9)##	62.6 (57.9 – 67.3)	63.5 (58.9 – 68.1)#	65.8 (62.1 – 69.6)	63.1 (58.9 – 67.3)#
Italy		n	1533	52	74	192	60
	Work ability	t1 mean (95% CI)	40.1 (39.9 – 40.4)	39.0 (37.1 – 40.8)	37.6 (36.3 – 39.0)***	39.4 (38.7 – 40.2)	39.3 (37.6 – 40.9)
		t2 mean (95% CI)	40.0 (39.8 – 40.3)	39.5 (37.9 – 41.0)	37.3 (35.9 – 38.8)##	39.4 (38.7 – 40.1)	40.4 (39.0 – 41.9)##
	General health	t1 mean (95% CI)	66.8 (65.9 – 67.7)	66.2 (60.5 – 71.8)	59.7 (54.5 – 65.0)***	64.5 (62.0 – 67.1)	64.3 (59.4 – 69.1)
		t2 mean (95% CI)	63.4 (62.5 – 64.3)###	64.4 (58.3 – 70.5)	57.5 (53.0 – 62.1)	62.3 (60.1 – 64.7)	65.7 (61.3 – 70.0)*
Netherlands		n	903	16	81	37	70
	Work ability	t1 mean (95% CI)	42.1 (41.8 – 42.4)	40.8 (37.7 – 43.9)	40.1 (38.8 – 41.4)**	41.3 (39.9 – 42.7)	41.0 (40.0 – 42.1)
		t2 mean (95% CI)	42.0 (41.7 – 42.3)	40.1 (36.4 – 43.7)*	40.7 (39.3 – 42.0)	40.4 (38.7 – 42.0)	42.0 (40.9 – 43.2)
	General health	t1 mean (95% CI)	72.6 (71.6 – 73.6)	73.4 (64.7 – 82.2)	71.2 (67.9 – 74.6)	70.5 (65.6 – 75.5)	74.4 (70.9 – 77.8)
		t2 mean (95% CI)	70.4 (69.4 – 71.4)###	63.4 (54.2 – 72.5)*	70.9 (67.5 – 74.2)	65.7 (60.6 – 70.8)	71.4 (67.7 – 75.2)
Poland		n	1338	22	18	60	32
	Work ability	t1 mean (95% CI)	37.8 (37.5 – 38.1)	35.5 (32.9 – 38.0)*	36.8 (34.3 – 39.4)	38.5 (37.2 – 39.8)	36.5 (34.0 – 39.0)
		t2 mean (95% CI)	37.2 (36.9 – 37.5)###	30.9 (28.3 – 33.5)*** ##	36.3 (32.8 – 39.7)	36.5 (35.0 – 38.0)#	36.3 (33.8 – 38.7)
	General health	t1 mean (95% CI)	55.8 (54.8 – 56.7)	48.4 (38.5 – 58.4)	58.3 (49.6 – 67.1)	55.4 (51.8 – 59.1)	58.6 (50.6 – 66.5)
		t2 mean (95% CI)	51.4 (50.5 – 52.2)###	39.8 (30.2 – 49.3)**	50.6 (42.7 – 58.4)	46.8 (43.2 – 50.4)* ###	50.0 (43.2 – 56.8)#
Slovakia		n	389	7	12	34	15
	Work ability	t1 mean (95% CI)	40.0 (39.6 – 40.5)	39.6 (34.8 – 44.3)	39.3 (36.8 – 41.9)	38.7 (37.2 – 40.0)	40.4 (37.6 – 43.2)
		t2 mean (95% CI)	39.8 (39.3 – 40.2)	39.4 (34.1 – 44.7)	40.0 (37.7 – 42.3)	38.6 (36.9 – 40.2)	40.3 (36.7 – 44.0)
	General health	t1 mean (95% CI)	56.7 (55.0 – 58.4)	63.6 (43.3 – 83.9)	55.4 (43.3 – 67.5)	55.7 (48.7 – 62.7)	52.3 (40.3 – 64.3)
		t2 mean (95% CI)	53.2 (51.5 – 54.9)###	50.0 (26.1 – 73.9)	53.8 (42.5 – 65.0)	51.2 (46.0 – 56.3)	51.5 (41.4 – 61.6)

*indicate significance levels of differences between the reference group “*No, I did not ask*” and the other shift change groups at t1 and t2, respectively, adjusted for age, gender, country (overall) *p < 0.05, **p < 0.01, ***p < 0.001.

#indicate significance levels of within group differences between t1 and t2 (time trends), assessed by GLM Repeated Measures, adjusted for age, gender, and country (overall).

#p < 0.05, ## p < 0.01, ### p < 0.001.

The time effects for WAI in the overall samples may be summarized by the observation that those groups whose wish was fulfilled had a beneficial WAI development and vice versa. Nurses, whose wish to change shift schedule was not fulfilled, had reported the lowest work ability at baseline already (WAI mean (M) = 37.6), and had an even lower work ability one year later (M = 36.6, Δ = 1.0, p < 0.001). The large group of nurses who had remained working in their initial shift schedule and had no intention to change shift schedule showed a small but significant decrease in work ability (M = t1: 39.6, t2: 39.3, Δ = -0.3, p < 0.001). The group of nurses who were - against their preference - forced by their organisation to change their shift schedule also showed a significant deterioration in work ability between t1 and t2 (M = t1: 39.1, t2: 38.4, Δ = -0.7, p < 0.001). Work ability remained stable for nurses who were allowed to change shift schedule in line with their own requests (M = t1: 38.6, t2: 38.8, Δ = 0.2, n.s.). Notably, for the group of nurses who accepted a shift schedule change imposed by the institution a significant increase in work ability between t1 and t2 was found (M = t1: 39.4, t2: 40.0, Δ = 0.6, p < 0.05).

Similar findings were observed for the different countries (Table 3). For example, in all countries apart from Italy (WAI change (Δ) by +0.5 points), the groups of nurses who in vain had asked for change of their shift schedule showed a decrease in work ability between t1 and t2. In all countries, with exception of Poland and Slovakia, the work ability of nurses who accepted a shift schedule change imposed by the institution ameliorated during the one year follow-up. This improvement in work ability was significant in France (Δ = +2.8; p < 0.05) and Italy (Δ = +1.1; p < 0.01). However, no significant changes in work ability for all five groups of shift schedule change were observed in the Netherlands and Slovakia. Perhaps this may be due to the small sample size of both countries.

### General health

The findings for general health were in line with those of WAI, the significance pattern, however, was different (Table 3). At baseline, none of the group differences with the reference group reached significance level. At t2 the two groups whose preference was not followed (“No, but I asked” and “Yes, institution imposed it”) had developed significantly lower mean scores for general health than the reference group.

Nurses who had no intention to change shift schedule and had remained working in their shift schedule had a significant but small decrease in general health (general health mean M = t1: 65.3, t2: 63.0, Δ = -2.3, p < 0.001), just like the group of those who were forced to change their shift schedule by their institution (M = t1: 63.8, t2: 60.7, Δ = -2.1, p < 0.001) – although on somewhat lower level. However, the level of general health remained rather stable among nurses who were allowed to change shift schedule as they requested (M = t1: 65.2, t2: 64.7, Δ = -0.5, n.s.) and among nurses who accepted a shift schedule change imposed by their institution (M = t1: 65.6, t2: 64.4, Δ = -1.1, n.s.). Generally, the group of nurses who was not allowed to change shift schedule although they had wished to do so, showed a sharp decline in perceived health one year later (M = t1: 63.9, t2: 59.2, Δ = 4.7, p < 0.001).

Considerable differences in general health scores were found between the participating countries, but the impact of shift schedule change on general health could more or less be observed in all of them. For example, for all eight countries, the level of general health decreased significantly between both measurements among those nurses who had no intention to change their shift schedule and remained working in their current shift schedule. In Germany nurses’ general health showed a significant increase among those nurses who were allowed by their institution to change their shift schedule as they requested (Δ = +2.2; p = < 0.05).

## Discussion

The objective of this secondary data longitudinal study was to examine if and how work ability and general health are associated with requested, forced and denied shift schedule changes among nurses. First of all, we found significant decreases in work ability and health in the reference group between t1 and t2. This may be in line with the ageing associated decrease, for work ability it lies within the expected decline [19]. Secondly, we found no decrease (not even the ageing related) for work ability and health among those who changed shift pattern in line with their will. Thirdly, we found a substantial decrease of work ability and health among those whose request for changing shift schedule was not followed and fourthly, we also find that this group already had low scores one year before responding to the shift change questions.

Our findings imply

– that a change of shift schedule may help to sustain nurses’ work ability and health if it occurs in line with the nurses’ wish,

– that imposing a shift schedule against the nurses’ preference (forced change or denial of requested change) may further decrease already low work ability and health, and

– work ability may be more sensitive to these processes than self-reported general health.

### Working time, health and work ability

With regard to the association of working time and general health, some studies from Finland, Sweden and Germany support our findings [20-22]. Ala-Mursula and colleagues [23,24], found that females have an increased risk of health problems when they have for a long time low control over their working time. Similar results were described in a study by Elovainio et al. [25], a lack of control over one’s working time was found to predict long-term sickness absence among females; the more uncertain employees are about their working time, the higher the risk. The effects are not only on work ability and general health, but also on disability pension [26].

However, our overall findings suggest that although the associations are comparable for both work ability and self-reported general health, a change in shift schedule has a larger effect on work ability than on general health. This indicates that among nurses, health and work ability may constitute distinct concepts which are affected by work exposure (such as shift schedules) differently. To transfer this to the nurses’ perception one may say that that nurses who are working shifts against their preference are more likely to state: “I can’t handle this any longer” (work ability) than stating: “this makes me sick” (health).

### Organizational consequences

The differentiation of work ability and health has consequences for the health care organization: The responsibility to improve work ability predominantly rests upon the organization and to somewhat lesser degree also on the employee (according to the model of work ability, Ilmarinen 1999). In contrast, the responsibility to promote one’s health rests to higher degree upon the individual employee given that health strongly depends on one’s lifestyle. However, organizations can contribute to the promotion of their workers’ health for instance within work place health promotion activities. Taking into account that a deterioration in work ability is a predictor of organizational and professional turnover intentions [27] a focus on the promotion of work ability is needed.

Forced and denied changes of shift schedule may have adverse effects on both workers‘ work ability and health. Health care organizations should thoroughly consider nurses’ shift preferences of their nursing employees and try to search for appropriate solutions. Forcing nurses to change their shift schedule should be avoided as much as possible. In case this is not possible, the decision for a forced change should be presented with reasonable explanations and the new shift schedule should be as much in line with the expectations of the employees as possible in order to sustain their work ability and health, and to prevent the professional turnover consequently.

### National differences

As for the classification of schedule change, we have to consider group differences of group sizes: the reference group constitutes 83% of the total sample, the adverse groups 2.3% (“No, but I asked”) and 5.6% (“Yes, institution imposed it”), respectively, and the positive groups 5.4% (“Yes, because I asked”) and 3.6% (“Yes, institution imposed it, I accepted”). However, we find considerable national differences. In the Netherlands, the adverse groups add up to 4.7% and the positive groups to 13.6% of the total sample. In contrast, the high proportions of adverse groups in the national samples of Slovakia (8.9%), Germany (9.6%), France (9.7%) and Italy (12.7%) may indicate a potential for using the beneficial impact of nurses’ participation in work scheduling in these countries. On the other hand, high proportions of nurses in the positive groups in the Netherlands (13.6%), Finland (12.6%) and France (12.5%) may indicate a participatory work culture in this respect.

### Strengths and limitations

A strength of this investigation is its’ focus on one distinct professional group, the large size, its’ longitudinal character and the inclusion of several countries allowing for cross cultural validation of findings. In fact, our analyses indicated that our findings were to high degree consistent across the participating countries.

On the other hand, the study also has several limitations which should be considered. First, the data is based on self-reports, meaning that an influence of common-method bias and recall bias on the observed associations may not be completely ruled out. According to P Spector [28] the influence of common method variance is often being overestimated and C Tennant [29] as well as T Theorell and HM Hasselhorn [30] emphasize that the use of self-report measures for both exposure and outcome variables is less problematic in a prospective design. However, we have bear in mind the overall response rates of baseline and follow-up surveys were moderate only.

Furthermore, sample attrition may have affected our results. The comparison between respondents and non-respondents suggest a lower general health and lower work ability of participants. The impact of this on the findings cannot be estimated.

In this study, we could not analyze the sample with respect to current (and past) shift schedules and their relation to shift preferences and shift changes. The sample of 11,102 nurses is too small for this analysis and national differences of shift pattern distributions might have influenced the findings. However, we regard the question for the nurses’ preference to change shift or not as a strength, as it may constitute a valid tool reflecting (the responding nurses’) individual needs and an easy to handle organizational indicator and managing tool. In addition, we did not conduct separate analysis for nurses working in different institutions due to the extremely small sample size in in some countries, particularly nurse working in nursing homes and home care services from the Netherlands and Slovakia.

A further limitation is certainly that our data and analyses reflect nursing work performed 10 years ago. In this analysis we mainly emphasize patterns (works schedule change preference and development of work ability and health) and only to lower degree conditions (comparison of mean values). Patterns may be assumed to be more stable than conditions. However, the increasing workload in the past decade among nurses in Europe [31,32] indicates that the patterns found deserve emphasis and further scientific and organizational attention.

Finally, one aspect needs to be considered when emphasizing work ability over health: The study has a one year follow-up period only. This means that potential time-lagged effects not covering this period cannot be observed – and the time lagged effects may be different for work ability than for health. It is possible that the effects of a forced or denied change of shift schedule has initially a shorter term effect on employees’ work ability but a longer term effect on their health, which cannot be measured the subsequent 12 months period. However, if a work ability effect precedes the health effect, then work ability may not only be an indicator for work organizational matters but also for future changes in workers’ health. Work ability may thus deserve even more attention in work organization in health care.

## Conclusions

The findings imply that shift schedule organization may constitute a valuable preventive tool to promote nurses’ work ability and – possibly to lesser extent – their health. The nurses’ shift preference may be considered not only as individual preventive measure but also as organisational a human resource management indicator. Research should also investigate longer time intervals than one year and further outcomes such as organizational and professional commitment and work participation.

## Abbreviations

NEXT: Nurses early exit study; WAI: Work ability index.

## Competing interests

The authors declare that they have no competing interests.

## Pre-publication history

The pre-publication history for this paper can be accessed here:

http://www.biomedcentral.com/1471-2458/13/1137/prepub
